# (*E*)-*N*′-(4-Chloro­benzyl­idene)-2-meth­oxy­benzohydrazide

**DOI:** 10.1107/S160053681300175X

**Published:** 2013-01-23

**Authors:** M. Syukri. Baharudin, Muhammad Taha, Nor Hadiani Ismail, Syed Adnan Ali Shah, Sammer Yousuf

**Affiliations:** a Atta-ur-Rahman Institute for Natural Product Discovery, Universiti Teknologi MARA (UiTM), Puncak Alam Campus, 42300 Bandar Puncak Alam, Selangor D. E. Malaysia; b Faculty of Applied Science, Universiti Teknologi MARA (UiTM), 40450 Shah Alam, Selangor D. E. Malaysia; c Department of Pharmacology and Chemistry, Faculty of Pharmacy, Universiti Teknologi MARA (UiTM), Puncak Alam Campus, 42300 Puncak Alam, Selangor D. E., Malaysia; d H.E.J. Research Institute of Chemistry, International Center for Chemical and Biological Sciences, University of Karachi, Karachi 75270, Pakistan

## Abstract

In the title hydrazone derivative, C_15_H_13_ClN_2_O_2_, the dihedral angle between the benzene rings is 2.36 (2)°. An intra­molecular N—H⋯O hydrogen bond is present. In the crystal, N—H⋯O and C—H⋯O hydrogen bonds link the mol­ecules into chains running parallel to the *b* axis.

## Related literature

For applications and biological activity of hydrazone derivatives, see: Khan *et al.* (2011 ▶, 2012 ▶); Kūçūkgūzel *et al.* (1999 ▶); Patel *et al.* (1984 ▶); Wilder (1967 ▶); Glasser & Doughty (1962 ▶). For a related structure, see: Cao (2009 ▶).
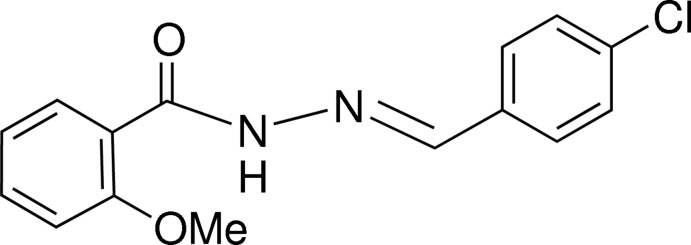


## Experimental

### 

#### Crystal data

C_15_H_13_ClN_2_O_2_
*M* *_r_* = 288.72
Orthorhombic, 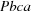

*a* = 12.5830 (7) Å
*b* = 9.8335 (5) Å
*c* = 23.6377 (13) Å
*V* = 2924.8 (3) Å^3^
*Z* = 8
Mo *K*α radiation
μ = 0.26 mm^−1^
*T* = 273 K
0.48 × 0.27 × 0.10 mm

#### Data collection

Bruker SMART APEX CCD area-detector diffractometer
Absorption correction: multi-scan (*SADABS*; Bruker, 2000 ▶) *T*_min_ = 0.884, *T*_max_ = 0.974
16194 measured reflections
2713 independent reflections
2021 reflections with *I* > 2σ(*I*)
*R*_int_ = 0.030

#### Refinement

*R*[*F* ^2^ > 2σ(*F* ^2^)] = 0.044
*wR*(*F* ^2^) = 0.124
*S* = 1.06
2713 reflections
186 parameters
H atoms treated by a mixture of independent and constrained refinement
Δρ_max_ = 0.32 e Å^−3^
Δρ_min_ = −0.35 e Å^−3^

### 

Data collection: *SMART* (Bruker, 2000 ▶); cell refinement: *SAINT* (Bruker, 2000 ▶); data reduction: *SAINT*; program(s) used to solve structure: *SHELXS97* (Sheldrick, 2008 ▶); program(s) used to refine structure: *SHELXL97* (Sheldrick, 2008 ▶); molecular graphics: *SHELXTL* (Sheldrick, 2008 ▶); software used to prepare material for publication: *SHELXTL*, *PARST* (Nardelli, 1995 ▶) and *PLATON* (Spek, 2009 ▶).

## Supplementary Material

Crystal structure: contains datablock(s) global, I. DOI: 10.1107/S160053681300175X/rz5038sup1.cif

Structure factors: contains datablock(s) I. DOI: 10.1107/S160053681300175X/rz5038Isup2.hkl

Supplementary material file. DOI: 10.1107/S160053681300175X/rz5038Isup3.cml

Additional supplementary materials:  crystallographic information; 3D view; checkCIF report

## Figures and Tables

**Table 1:** Hydrogen-bond geometry (Å, °)

*D*—H⋯*A*	*D*—H	H⋯*A*	*D*⋯*A*	*D*—H⋯*A*
N1—H1*A*⋯O2	0.76 (2)	2.04 (2)	2.632 (2)	135.7 (19)
N1—H1*A*⋯O1^i^	0.76 (2)	2.58 (2)	3.163 (3)	135.5 (19)
C8—H8*A*⋯O1^i^	0.93	2.42	3.127 (3)	132

Symmetry code: (i) 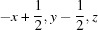.

**Fig. 1.**
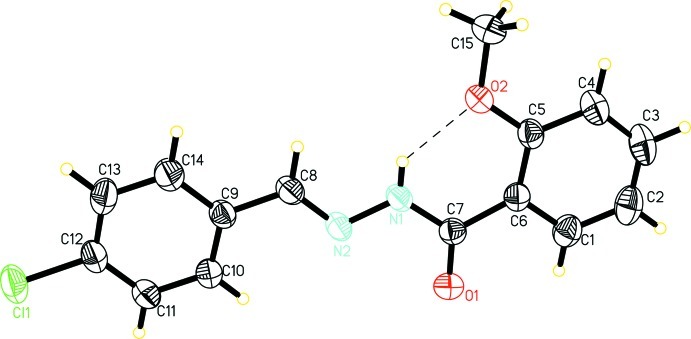
The molecular structure of the title compound with displacement ellipsoids drawn at 30% probability level. An intramolecular hydrogen bond is shown as a dashed line.

**Fig. 2.**
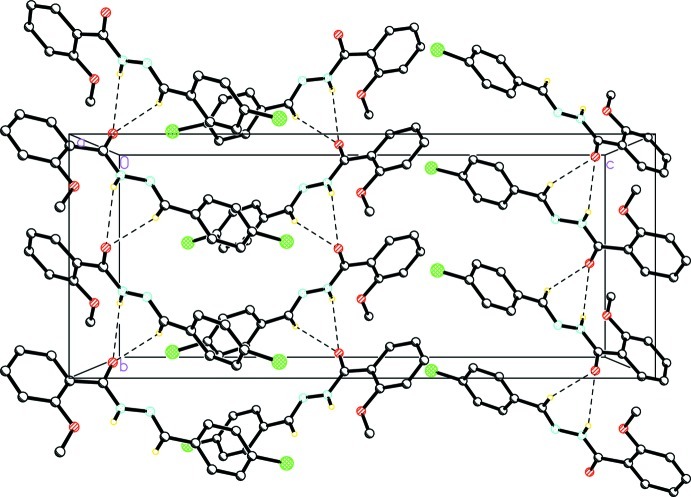
Crystal packing of the title compound viewed down the a axis. Only hydrogen atoms involved in hydrogen bonding are shown.

## supplementary crystallographic information

### Comment

Organic compounds based on the hydrazone moiety are well known due to their wide range of applications both in structural and medicinal chemistry (Khan *et al.*, 2011, 2012; Kūçūkgūzel *et al.*, 1999; Patel *et al.*, 1984; Wilder, 1967; Glasser & Doughty, 1962). The title compound is a hydrazone derivative synthesized in order to evaluate its biological activities.

The structure of title compound (Fig. 1) is similar to that of the previously published compound (*E*)-*N*'-(2-chlorobenzylidene)-2-methoxybenzohydrazide (Cao, 2009) with the difference that the 2-chlorobenzene ring is replaced by a 4-chlorobenzene ring (C9–19 C14). The bond lengths and angles were found to be similar to those observed in the structurally related phenyl hydrazone (Cao, 2009). The azomethine double bond adopts an *E* configuration (C═N, 1.270 (3) Å). The molecular conformation is stablized by an intramolecular N1—H1A···O2 hydrogen bond (Table 1) to generate an *S*6 graph set ring motif. N1—H1A···O1 and C8—H8A···O1 hydrogen bonds play important roles in stabilizing the crystal structure by forming chains running parallel to the *b* axis (Fig. 2).

### Experimental

The title compound was synthesized by refluxing in methanol a mixture of 2-methoxybenzohydrazide (0.332 g, 2 mmol), 4-chlorobenzaldehyde (0.281 g, 2 mmol) and a catalytical amount of acetic acid for 3 h. The progress of reaction was monitored by TLC. After completion of the reaction, the solvent was evaporated by vacuum to afford the crude product which was further recrystallized in methanol to obtain colourless crystals (0.467 g, 81% yield). All chemicals were purchased by sigma Aldrich, Germany.

### Refinement

H atoms on methyl, phenyl and methine carbon atoms were positioned geometrically with C—H = 0.96 (CH_3_) and 0.93 Å (CH) and constrained to ride on their parent atoms with *U*_iso_(H) = 1.5*U*_eq_(CH_3_) or 1.2*U*_eq_(CH). The H atoms on the nitrogen (N–H = 0.76 (2) Å) and oxygen (O–H = 0.84 (2)–0.93 (2) Å) atoms were located in a difference Fourier map and refined isotropically. A rotating group model was applied to the methyl group.

### Crystal data

**Table:**

C_15_H_13_ClN_2_O_2_	*F*(000) = 1200
*M**_r_* = 288.72	*D*_x_ = 1.311 Mg m^−^^3^
Orthorhombic, *P**b**c**a*	Mo *K*α radiation, λ = 0.71073 Å
Hall symbol: -P 2ac 2ab	Cell parameters from 3504 reflections
*a* = 12.5830 (7) Å	θ = 2.4–24.1°
*b* = 9.8335 (5) Å	µ = 0.26 mm^−^^1^
*c* = 23.6377 (13) Å	*T* = 273 K
*V* = 2924.8 (3) Å^3^	Block, colourless
*Z* = 8	0.48 × 0.27 × 0.10 mm

### Data collection

**Table:**

Bruker SMART APEX CCD area-detector diffractometer	2713 independent reflections
Radiation source: fine-focus sealed tube	2021 reflections with *I* > 2σ(*I*)
Graphite monochromator	*R*_int_ = 0.030
ω scan	θ_max_ = 25.5°, θ_min_ = 2.4°
Absorption correction: multi-scan (*SADABS*; Bruker, 2000)	*h* = −14→15
*T*_min_ = 0.884, *T*_max_ = 0.974	*k* = −11→11
16194 measured reflections	*l* = −28→28

### Refinement

**Table:**

Refinement on *F*^2^	Primary atom site location: structure-invariant direct methods
Least-squares matrix: full	Secondary atom site location: difference Fourier map
*R*[*F*^2^ > 2σ(*F*^2^)] = 0.044	Hydrogen site location: inferred from neighbouring sites
*wR*(*F*^2^) = 0.124	H atoms treated by a mixture of independent and constrained refinement
*S* = 1.06	*w* = 1/[σ^2^(*F*_o_^2^) + (0.0533*P*)^2^ + 0.7724*P*] where *P* = (*F*_o_^2^ + 2*F*_c_^2^)/3
2713 reflections	(Δ/σ)_max_ = 0.001
186 parameters	Δρ_max_ = 0.32 e Å^−^^3^
0 restraints	Δρ_min_ = −0.35 e Å^−^^3^

### Special details

**Table:**

Geometry. All e.s.d.'s (except the e.s.d. in the dihedral angle between two l.s. planes) are estimated using the full covariance matrix. The cell e.s.d.'s are taken into account individually in the estimation of e.s.d.'s in distances, angles and torsion angles; correlations between e.s.d.'s in cell parameters are only used when they are defined by crystal symmetry. An approximate (isotropic) treatment of cell e.s.d.'s is used for estimating e.s.d.'s involving l.s. planes.
Refinement. Refinement of *F*^2^ against ALL reflections. The weighted *R*-factor *wR* and goodness of fit *S* are based on *F*^2^, conventional *R*-factors *R* are based on *F*, with *F* set to zero for negative *F*^2^. The threshold expression of *F*^2^ > σ(*F*^2^) is used only for calculating *R*-factors(gt) *etc*. and is not relevant to the choice of reflections for refinement. *R*-factors based on *F*^2^ are statistically about twice as large as those based on *F*, and *R*- factors based on ALL data will be even larger.

### Fractional atomic coordinates and isotropic or equivalent isotropic displacement parameters (Å^2^)

**Table:**

	*x*	*y*	*z*	*U*_iso_*/*U*_eq_	
Cl1	0.01370 (6)	0.06519 (7)	0.64198 (3)	0.0891 (3)	
O1	0.18055 (13)	0.53482 (16)	0.95365 (6)	0.0713 (4)	
O2	0.43399 (11)	0.28997 (14)	0.99795 (6)	0.0659 (4)	
N1	0.27223 (15)	0.3462 (2)	0.93138 (7)	0.0582 (5)	
H1A	0.3141 (16)	0.295 (2)	0.9402 (8)	0.048 (6)*	
N2	0.21338 (13)	0.32469 (18)	0.88327 (7)	0.0578 (5)	
C1	0.2839 (2)	0.5735 (2)	1.05402 (9)	0.0710 (6)	
H1B	0.2255	0.6267	1.0443	0.085*	
C2	0.3375 (2)	0.5997 (3)	1.10386 (11)	0.0896 (8)	
H2B	0.3155	0.6702	1.1274	0.108*	
C3	0.4228 (3)	0.5217 (4)	1.11831 (11)	0.0981 (9)	
H3A	0.4587	0.5393	1.1519	0.118*	
C4	0.4567 (2)	0.4175 (3)	1.08405 (10)	0.0800 (7)	
H4A	0.5150	0.3652	1.0945	0.096*	
C5	0.40351 (16)	0.3906 (2)	1.03376 (8)	0.0562 (5)	
C6	0.31540 (16)	0.4690 (2)	1.01800 (8)	0.0536 (5)	
C7	0.25085 (17)	0.4537 (2)	0.96504 (8)	0.0532 (5)	
C8	0.23811 (16)	0.2194 (2)	0.85504 (8)	0.0607 (6)	
H8A	0.2935	0.1645	0.8675	0.073*	
C9	0.18113 (16)	0.1828 (2)	0.80327 (8)	0.0567 (5)	
C10	0.09208 (18)	0.2521 (2)	0.78495 (9)	0.0645 (6)	
H10A	0.0663	0.3246	0.8062	0.077*	
C11	0.04081 (19)	0.2156 (2)	0.73582 (9)	0.0678 (6)	
H11A	−0.0191	0.2632	0.7240	0.081*	
C12	0.07826 (18)	0.1091 (2)	0.70448 (8)	0.0645 (6)	
C13	0.1646 (2)	0.0373 (3)	0.72182 (11)	0.0889 (8)	
H13A	0.1889	−0.0359	0.7006	0.107*	
C14	0.2162 (2)	0.0741 (3)	0.77145 (11)	0.0843 (8)	
H14A	0.2751	0.0247	0.7834	0.101*	
C15	0.51850 (19)	0.2009 (2)	1.01362 (11)	0.0751 (7)	
H15A	0.5257	0.1307	0.9857	0.113*	
H15B	0.5031	0.1606	1.0497	0.113*	
H15C	0.5836	0.2515	1.0160	0.113*	

### Atomic displacement parameters (Å^2^)

**Table:**

	*U*^11^	*U*^22^	*U*^33^	*U*^12^	*U*^13^	*U*^23^
Cl1	0.1105 (6)	0.0983 (6)	0.0586 (4)	−0.0205 (4)	−0.0205 (3)	−0.0091 (3)
O1	0.0817 (11)	0.0609 (10)	0.0711 (10)	0.0121 (8)	−0.0116 (8)	0.0011 (7)
O2	0.0701 (9)	0.0635 (10)	0.0640 (9)	0.0090 (7)	−0.0198 (7)	−0.0044 (8)
N1	0.0554 (11)	0.0685 (12)	0.0506 (10)	0.0102 (10)	−0.0114 (8)	−0.0033 (9)
N2	0.0566 (10)	0.0696 (12)	0.0472 (9)	0.0031 (8)	−0.0080 (7)	−0.0016 (8)
C1	0.0811 (16)	0.0689 (15)	0.0631 (13)	−0.0042 (12)	0.0063 (11)	−0.0067 (11)
C2	0.109 (2)	0.092 (2)	0.0677 (15)	−0.0094 (17)	0.0051 (15)	−0.0277 (14)
C3	0.109 (2)	0.122 (2)	0.0634 (16)	−0.011 (2)	−0.0229 (15)	−0.0227 (17)
C4	0.0838 (16)	0.0930 (19)	0.0632 (14)	−0.0032 (14)	−0.0199 (12)	−0.0054 (13)
C5	0.0597 (12)	0.0579 (13)	0.0510 (11)	−0.0119 (10)	−0.0057 (9)	0.0025 (10)
C6	0.0600 (12)	0.0528 (12)	0.0481 (10)	−0.0093 (10)	0.0017 (9)	0.0026 (9)
C7	0.0565 (12)	0.0529 (12)	0.0501 (11)	−0.0034 (10)	0.0005 (9)	0.0048 (9)
C8	0.0565 (12)	0.0751 (15)	0.0504 (11)	0.0103 (11)	−0.0054 (9)	0.0000 (11)
C9	0.0601 (12)	0.0651 (13)	0.0449 (10)	0.0040 (10)	−0.0014 (9)	0.0003 (9)
C10	0.0707 (14)	0.0663 (14)	0.0564 (12)	0.0058 (11)	−0.0077 (10)	−0.0072 (10)
C11	0.0705 (14)	0.0718 (15)	0.0610 (13)	0.0018 (12)	−0.0149 (11)	0.0016 (11)
C12	0.0735 (14)	0.0731 (15)	0.0468 (11)	−0.0122 (12)	−0.0056 (10)	0.0004 (10)
C13	0.1018 (19)	0.095 (2)	0.0701 (15)	0.0180 (16)	−0.0084 (14)	−0.0314 (14)
C14	0.0852 (17)	0.097 (2)	0.0708 (15)	0.0272 (15)	−0.0162 (13)	−0.0182 (14)
C15	0.0708 (15)	0.0766 (17)	0.0778 (16)	0.0116 (12)	−0.0149 (12)	0.0070 (13)

### Geometric parameters (Å, º)

**Table:**

Cl1—C12	1.740 (2)	C5—C6	1.401 (3)
O1—C7	1.221 (2)	C6—C7	1.500 (3)
O2—C5	1.357 (2)	C8—C9	1.463 (3)
O2—C15	1.427 (2)	C8—H8A	0.9300
N1—C7	1.350 (3)	C9—C14	1.380 (3)
N1—N2	1.374 (2)	C9—C10	1.381 (3)
N1—H1A	0.76 (2)	C10—C11	1.376 (3)
N2—C8	1.270 (3)	C10—H10A	0.9300
C1—C2	1.382 (3)	C11—C12	1.366 (3)
C1—C6	1.392 (3)	C11—H11A	0.9300
C1—H1B	0.9300	C12—C13	1.359 (3)
C2—C3	1.363 (4)	C13—C14	1.389 (3)
C2—H2B	0.9300	C13—H13A	0.9300
C3—C4	1.374 (4)	C14—H14A	0.9300
C3—H3A	0.9300	C15—H15A	0.9600
C4—C5	1.390 (3)	C15—H15B	0.9600
C4—H4A	0.9300	C15—H15C	0.9600
			
C5—O2—C15	119.75 (16)	N2—C8—H8A	119.3
C7—N1—N2	120.07 (19)	C9—C8—H8A	119.3
C7—N1—H1A	119.8 (16)	C14—C9—C10	118.1 (2)
N2—N1—H1A	119.9 (16)	C14—C9—C8	119.3 (2)
C8—N2—N1	115.37 (18)	C10—C9—C8	122.58 (19)
C2—C1—C6	121.3 (2)	C11—C10—C9	121.1 (2)
C2—C1—H1B	119.3	C11—C10—H10A	119.5
C6—C1—H1B	119.3	C9—C10—H10A	119.5
C3—C2—C1	119.5 (3)	C12—C11—C10	119.7 (2)
C3—C2—H2B	120.2	C12—C11—H11A	120.1
C1—C2—H2B	120.2	C10—C11—H11A	120.1
C2—C3—C4	121.1 (2)	C13—C12—C11	120.7 (2)
C2—C3—H3A	119.5	C13—C12—Cl1	120.00 (19)
C4—C3—H3A	119.5	C11—C12—Cl1	119.31 (18)
C3—C4—C5	119.8 (3)	C12—C13—C14	119.6 (2)
C3—C4—H4A	120.1	C12—C13—H13A	120.2
C5—C4—H4A	120.1	C14—C13—H13A	120.2
O2—C5—C4	122.5 (2)	C9—C14—C13	120.8 (2)
O2—C5—C6	117.33 (17)	C9—C14—H14A	119.6
C4—C5—C6	120.2 (2)	C13—C14—H14A	119.6
C1—C6—C5	118.0 (2)	O2—C15—H15A	109.5
C1—C6—C7	115.5 (2)	O2—C15—H15B	109.5
C5—C6—C7	126.51 (19)	H15A—C15—H15B	109.5
O1—C7—N1	121.7 (2)	O2—C15—H15C	109.5
O1—C7—C6	120.70 (19)	H15A—C15—H15C	109.5
N1—C7—C6	117.56 (19)	H15B—C15—H15C	109.5
N2—C8—C9	121.32 (19)		
			
C7—N1—N2—C8	−178.62 (19)	C5—C6—C7—O1	−174.3 (2)
C6—C1—C2—C3	−0.2 (4)	C1—C6—C7—N1	−174.05 (19)
C1—C2—C3—C4	0.2 (5)	C5—C6—C7—N1	6.8 (3)
C2—C3—C4—C5	0.0 (4)	N1—N2—C8—C9	179.13 (18)
C15—O2—C5—C4	4.9 (3)	N2—C8—C9—C14	174.7 (2)
C15—O2—C5—C6	−175.69 (19)	N2—C8—C9—C10	−6.1 (3)
C3—C4—C5—O2	179.3 (2)	C14—C9—C10—C11	−1.2 (3)
C3—C4—C5—C6	−0.2 (4)	C8—C9—C10—C11	179.5 (2)
C2—C1—C6—C5	0.1 (3)	C9—C10—C11—C12	0.0 (4)
C2—C1—C6—C7	−179.1 (2)	C10—C11—C12—C13	1.1 (4)
O2—C5—C6—C1	−179.35 (18)	C10—C11—C12—Cl1	−179.24 (18)
C4—C5—C6—C1	0.1 (3)	C11—C12—C13—C14	−1.0 (4)
O2—C5—C6—C7	−0.2 (3)	Cl1—C12—C13—C14	179.3 (2)
C4—C5—C6—C7	179.2 (2)	C10—C9—C14—C13	1.3 (4)
N2—N1—C7—O1	−0.9 (3)	C8—C9—C14—C13	−179.4 (2)
N2—N1—C7—C6	177.96 (17)	C12—C13—C14—C9	−0.2 (4)
C1—C6—C7—O1	4.8 (3)		

### Hydrogen-bond geometry (Å, º)

**Table:**

*D*—H···*A*	*D*—H	H···*A*	*D*···*A*	*D*—H···*A*
N1—H1*A*···O2	0.76 (2)	2.04 (2)	2.632 (2)	135.7 (19)
N1—H1*A*···O1^i^	0.76 (2)	2.58 (2)	3.163 (3)	135.5 (19)
C8—H8*A*···O1^i^	0.93	2.42	3.127 (3)	132

Symmetry code: (i) −*x*+1/2, *y*−1/2, *z*.
